# Crowdsourced Identification of Possible Allergy-Associated Factors: Automated Hypothesis Generation and Validation Using Crowdsourcing Services

**DOI:** 10.2196/resprot.5851

**Published:** 2017-05-16

**Authors:** Eiji Aramaki, Shuko Shikata, Satsuki Ayaya, Shin-Ichiro Kumagaya

**Affiliations:** ^1^ Social Computing Lab Graduate School of Information Science Nara Institute of Science and Technology Ikoma Japan; ^2^ Research Center for Advanced Science and Technology University of Tokyo Tokyo Japan

**Keywords:** allergy, crowdsourcing, disease risk, automatic abduction, Tohjisha-Kenkyu, self-support study

## Abstract

**Background:**

Hypothesis generation is an essential task for clinical research, and it can require years of research experience to formulate a meaningful hypothesis. Recent studies have endeavored to apply crowdsourcing to generate novel hypotheses for research. In this study, we apply crowdsourcing to explore previously unknown allergy-associated factors.

**Objective:**

In this study, we aimed to collect and test hypotheses of unknown allergy-associated factors using a crowdsourcing service.

**Methods:**

Using a series of questionnaires, we asked crowdsourcing participants to provide hypotheses on associated factors for seven different allergies, and validated the candidate hypotheses with odds ratios calculated for each associated factor. We repeated this abductive validation process to identify a set of reliable hypotheses.

**Results:**

We obtained two primary findings: (1) crowdsourcing showed that 8 of the 13 known hypothesized allergy risks were statically significant; and (2) among the total of 157 hypotheses generated by the crowdsourcing service, 75 hypotheses were statistically significant allergy-associated factors, comprising the 8 known risks and 53 previously unknown allergy-associated factors. These findings suggest that there are still many topics to be examined in future allergy studies.

**Conclusions:**

Crowdsourcing generated new hypotheses on allergy-associated factors. In the near future, clinical trials should be conducted to validate the hypotheses generated in this study.

## Introduction

This study aims to generate hypotheses for clinical research by querying the general public through a crowdsourcing service, which is one of the new services and research styles that have emerged with advances in information and communication technology. Hypothesis generation is one of the most essential tasks for clinical research. A good hypothesis can bring about insightful and applicable results, whereas an unreasonable hypothesis may not only hinder research efforts, but also waste time and money. As it can require years of experience to produce a single meaningful research hypothesis, it is usually the *experts*, such as medical researchers and clinical doctors, who usually propose hypotheses for study. However, it has recently been suggested that hypotheses remain that even these experts have yet to address [1]. Applying the knowledge of the general public and the wisdom of crowds has drawn attention as a means to delve into these unexplored themes. This study does not provide an ultimate cost- and labor-cutting means for removing inadequate candidate hypotheses. However, the aim of the study is to acquire broader and more flexible possibilities of detecting possible novel risk factors for allergies with very quick and costless means, which would take much more money and time if practiced in traditional manners. This approach may also reduce the first step of validation by calculating odds ratios (ORs). Thus, this study is not intended to revise the traditional approach to research, but is capable of adding and accelerating the research of allergies with little cost and effort. We must note that for the final and concrete validation of hypotheses, we still require traditional medical diagnoses.

In this study, we investigated possible allergy-associated factors from hypothesis generation to questionnaire-based validation. An allergy occurs when a person's immune system reacts to substances in the environment that are harmless to most people. These substances are known as allergens and are found in house dust mites, pets, pollen, insects, foods, and some medicines. It has been estimated that approximately 1 of every 3 people in Japan suffers from some type of allergy, and this number is thought to be increasing [2]. According to a government research survey [3], 35.9% of survey respondents reported experiencing allergy symptoms during the year of the survey; among those, 14.7% were diagnosed by a physician as having an allergic reaction. This result indicates that less than half of the people who claimed to experience allergy symptoms actually sought treatment from a physician. The survey results also showed that respondents wanted more reliable information about dealing with allergies.

The causes of allergies can be classified into two general categories: (1) the patient’s genetic factors, and (2) environmental factors [4]. Genetic factors include the patient’s gender, race, age, and perhaps most importantly, their hereditary determinants. However, the recent increases to the number of allergic disorders cannot be explained by genetic factors alone [5]. Other causes may include major environmental factors such as air pollution, allergen levels, dietary changes, as well as exposure to infectious diseases during early childhood. Sifting through the vast numbers of different possible environmental factors places a heavy burden on researchers when identifying allergy-associated factors, but this task may be suitable for crowdsourcing research. In this paper, we call a factor that has a causal relation to the allergy a *risk factor*. Cases in which the causal relation is ambiguous, we call the factor an *associated factor*. Many allergy sufferers have to work to manage their allergies throughout their lives, and they can become well-informed on their own allergies in the process.

Existing epidemiologic literature indicates that the standard process of disease risk studies usually follows two phases. First, a researcher identifies a possible associated factor (hypothesis generation phase). Second, more experts analyze statistical data to shed light on the relationship between the candidate risk factor and the target disease, using methods such as interventions, observations, and questionnaire investigations (hypothesis validation phase). In contrast, crowdsourcing-based studies conduct both the hypothesis generation phase and the validation phase relying only on efforts by the general public, with little input from experts and their knowledge. This approach can substantially reduce the costs, time, and effort required by more conventional research methods.

In this study, we have developed a questionnaire consisting of three sections. The first section addressed the presence or absence of allergy symptoms in the respondent. The second section asked questions regarding known allergy risk factors, such as, “*Do you wear piercings?*” The third section was specifically designed for hypothesis generation, and respondents were asked to post new hypotheses using the question, “*Please submit some of your own questions that you feel may help to detect the cause of an allergy. Were/Do/Did you ____?*” The new hypotheses provided by the participants were then included as new risk question candidates (in the second section) for the subsequent round of the questionnaire. Repeating this cycle enabled automatic hypothesis generation and validation.

Crowdsourced hypothesis generation is a creative production task that is distinct from many previous crowdsourcing tasks for clinical or medical studies. Many previous crowdsourcing studies have requested the public to fulfill a relatively simple task [6,7]. These tasks have included crowdsourcing-based endoscopic video image annotation [8], medical document annotation for information retrieval [9], and evaluation of a surgical operation performance [10,11]. However, a few studies have already undertaken the challenge of generating medical hypotheses via crowdsourcing services. At present, new risk hypotheses for obesity [12,13], eczema [14], and acne treatment [15] have been generated through crowdsourcing-based procedures. Our study offers the following three novel features that distinguish it from previous studies:

This paper proposes a new methodology for crowdsourcing service-based risk research.

Unlike the diseases addressed in previous studies [12-14], allergies (the target subject of this study) covers a broad range of diseases. Simultaneously focusing on multiple diseases is a novel feature in our study.

While several previous studies have developed and used original crowdsourcing services, this study utilizes a standard commercially available Web service (Yahoo! Crowdsourcing Service [16]).

## Methods

### Ethics Statement

All participants provided written informed consent before participating in this study, and agreed to the terms of the Ethics Statement provided by Yahoo! Japan crowdsourcing service when they proceeded to the task page. All participants were informed of the aim of the questionnaire, and were told that their responses could be published in the future as part of a research study.

This study did not require the participants to be involved in any physical and/or mental intervention. Participants’ information was unlinkable, anonymized, and deidentified prior to analysis. This research did not obtain identifiable private information, meaning that it was exempt from Institutional Review Board approval according to the Ethical Guidelines for Research of the Japanese national government.

### Materials

Most materials for this study were gathered through crowdsourcing. However, 24 known allergy risk factors were initially cited from a previous study [17] and used as seed questions. Sample questions of our study questionnaire (hereinafter referred to as *questions*) are listed in Multimedia Appendix 1. The questionnaire consisted of 3 types of questions: (1) profile-related questions (*profile questions*); (2) risk questions, including known risks (*risk questions*); and (3) a question asking participants to propose questions regarding novel allergy risk factors (*novel risk-proposal questions*). We repeated the crowdsourcing process for 5 iterations (rounds), and the number of risk questions and their content were modified after each round as the hypotheses increased and evolved due to the crowdsourcing procedures.

#### Profile Questions

This section comprised questions regarding the basic profile of the participants, such as their allergy status, gender, and age. In this study, we examined the following allergy types: asthma, pollinosis, allergic rhinitis, atopic dermatitis, food allergy, drug hypersensitivity, and sick building syndrome. Pollinosis is regarded as a subgroup of allergic rhinitis. In the questionnaire, however, we divided these two concepts as different diseases so that the crowdworkers could easily understand. We investigated each of the aforementioned allergies independently, as well as in total (as some participants had more than one allergy type).

#### Risk Questions

This section comprised questions regarding each participant’s environmental (and partly genetic) situations. The initial risk questions consisted of 8 known risks (randomly selected from 24 known risks), which were used as seed questions. The questions in the second and third rounds contained both seed questions and the newly proposed questions by participants. The ORs for each associated factor and allergy (7 different allergies and overall) were estimated.

#### Novel Risk-Proposal Question

We asked the participants to suggest novel hypotheses for proposing associated factors in the form of questions, which were provided as risk questions in the subsequent round. Some of these questions were hard to match with the known risk factors of previous studies; we refer to such factors as *associated factors* in this study. At least one answer was required, with a maximum of five answers accepted

### Participants

All participants were recruited via Yahoo! Crowdsourcing Service. A total of 502 adults (303 men, 199 women) aged 20-69 years participated in this study, and their allergy types are shown in Table 1. The approximate sample size (n=500) was chosen according to a previous study [13], which gathered 532 samples via a crowdsourcing service.

**Table 1:** Number of participants for each allergy type (some having more than one type).

Allergy Type	Reported Prevalence Rate	Allergy, n (%)
Total (n=502)	50% [18]	298 (59.4%)
Asthma	5.4% [18]	38 (7.6%)
Pollinosis	26.5% (particularly for cedar pollen) [18]	183 (36.5%)
Allergic rhinitis	39.4% [19]	130 (25.9%)
Atopic dermatitis	9.4% (age: 20s), 8.3% (age: 30s), 4.8% (age: 40s) [18]	59 (11.8%)
Food allergy	Insufficient sampling surveys [18]	26 (5.2%)
Drug hypersensitivity	Insufficient sampling surveys [18]	13 (2.6%)
Sick building syndrome	Insufficient sampling surveys [20]	12 (2.4%)

### Procedure

**Figure 1:**
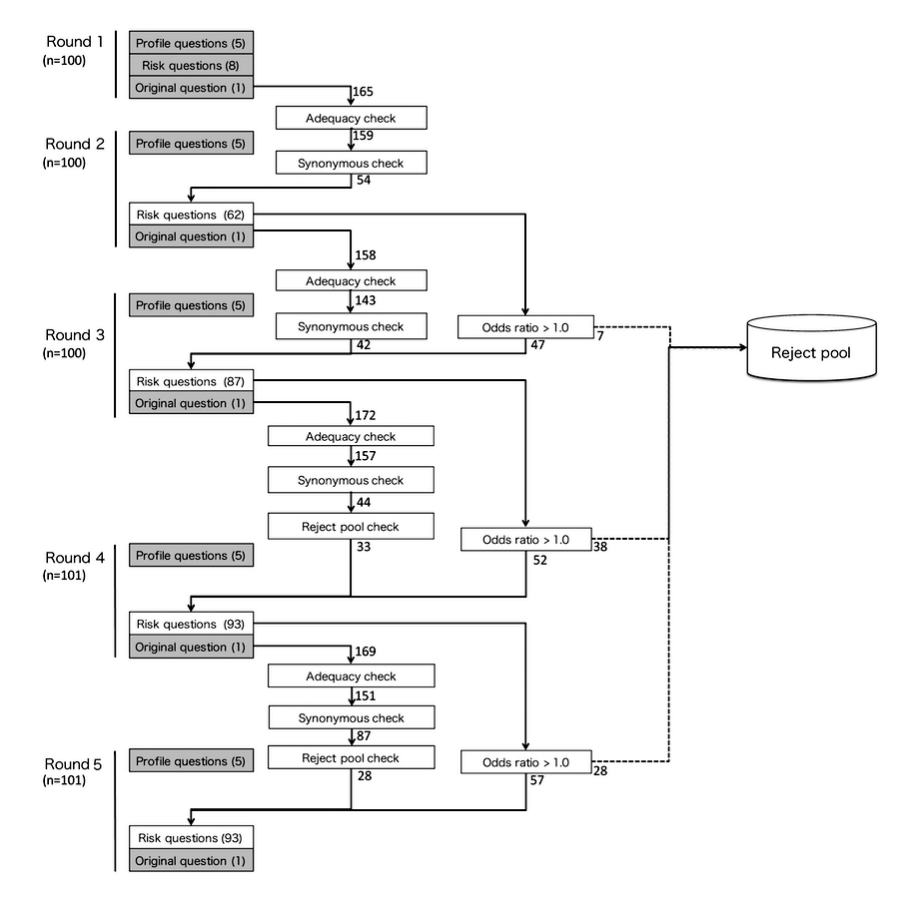
Experiment flow.

Figure 1 illustrates the crowdsourcing process. We repeated this process five times. Notices were posted on the Yahoo! Crowdsourcing job offer site [16] in both the *Easy Task* category and the category for those without particular professional skills.

After running an iteration of the procedure, we calculated the ORs of each answer for all risk questions. Based on these ORs, only the potentially promising top 99 (or fewer, if not applicable) questions of that round were retained for the subsequent round as risk factor questions; questions whose answers failed to score adequately high ORs were discarded. The detailed procedures are listed in Textbox 1. For the newly created questions provided by the participants in Round 1 that appeared suitable, we manually sorted these questions and combined similar questions (synonymous check) to make them more general. We also selected a candidate question if it was more general than other similar questions, and discarded the others (eg, when the questions were, “*Do you have a cat?*” and, “*Do you have pets?*” we discarded the former question and kept the latter).

**Procedure for the generation of research questions.**

**Round 1**: We asked 5 profile questions, 8 seed questions, and a question in which participants were required to provide 1 to 5 hypotheses of possible risks or causes of allergies (in a format that was similar to that of the 8 seed questions).

**Round 2**: We calculated the ORs of the answers for the 8 seed questions. From this round, we utilized the novel risk-proposal questions (ie, hypotheses) provided by the participants. The answers to these questions were manually filtered to eliminate inadequate questions. Questions were determined to be inadequate if they fulfilled either of the following criteria:

- A question that did not require a “yes” or “no” response (eg, “*How many cats do you have?*”)
- A question that required participants to divulge personal information (eg, requires an answer such as “*I work at the XX Company.*”)

**Round 3**: This round was similar to Round 2, but the ORs of the newly proposed hypotheses, as well as those of the seed questions (known risks), were calculated.

**Rounds 4 and 5**: These rounds were similar to Round 3, and were conducted to validate the crowdsourced hypotheses.

## Results

### Allergy Distribution

A participant was classified as *allergy negative* when he or she checked the *none* box in Profile Question 5, and classified as *allergy positive* in the other cases. Allergy negative or positive fully depends on the crowdsourcing participants’ decision; false negative or false positive results are sometimes included in the results. As shown in Table 1, 298 of the 502 participants (59.4%) reported having allergies, which is slightly higher than government-reported estimates [3]. There were 38 participants (38/502, 7.6%) with asthma and 183 participants (183/502, 36.5%) with pollinosis, both of which are higher than government statistics [3]. In contrast, there were 130 participants (130/502, 25.9%) with allergic rhinitis, which was lower than the government estimate of 39.4% [3]. The number of participants with atopic dermatitis was 59 (59/502, 11.8%), which was above the government estimate for adults [3]. With regard to the other allergy types (food allergy, drug hypersensitivity, and sick building syndrome), there was insufficient statistical data from previous studies. In our study, there were 26 participants (26/502, 5.2%) with food allergies, 13 participants (13/502, 2.6%) with drug hypersensitivities, and 12 participants (12/502, 2.4%) with sick building syndrome.

### Hypotheses Generation

A total of 157 new hypotheses were proposed from the five-round crowdsourcing procedure; from these, 75 hypotheses showed significant ORs, as shown in Multimedia Appendix 2 A. Hypotheses were regarded as significant if the lower limits of their 95% confidence intervals were >1.0. Approximately 22% of the participants took part in multiple rounds. The participants of multiple rounds are identified by their identification numbers in the Yahoo! Crowdsourcing Service.

### Hypotheses Evaluation

**Figure 2:**
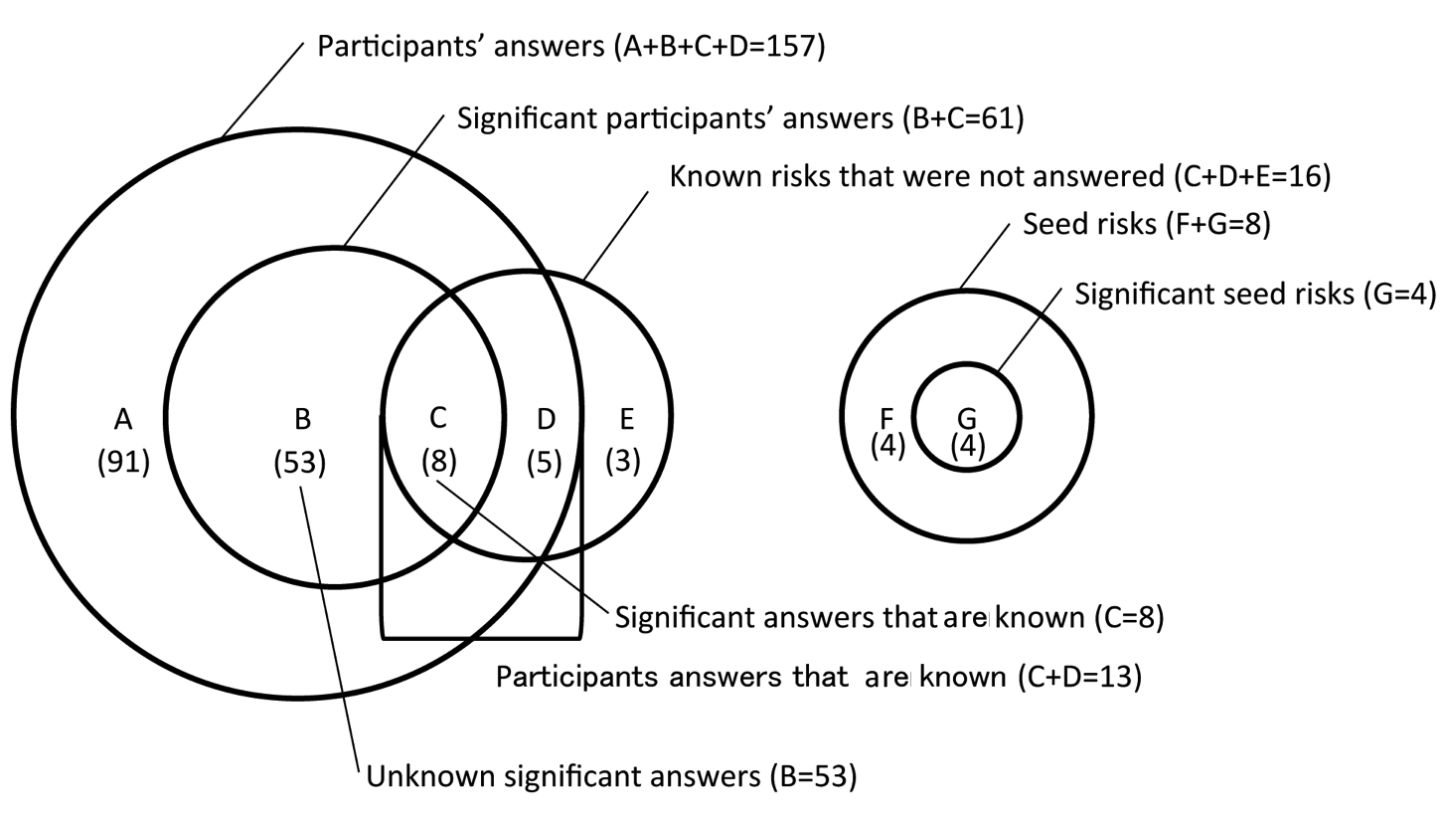
Relationship between participants’ answers and known risks.

The evaluation of these hypotheses was difficult because the aim of this study was to identify new risks. When a new candidate associated factor was initially identified, we were unable to evaluate it immediately, as its validity was still unclear. Therefore, instead of evaluating new associated factors, we evaluated the performance of reidentified ratio of the known risks. These known risks were identified from a review study [17] and government guidelines on allergies [21]. The relationship between participant answers and known risks are illustrated in Figure 2. By using these relationships, we evaluated the results for the following four aspects: Rediscovered Known Risk Ratio (RKRR), Significant Known Risks Ratio (SKRR), Significant Seed Risks Ratio (SSRR), and Significant Unknown Answer Ratio (SUAR).

#### Rediscovered Known Risk Ratio

The RKRR is the ratio of known risk factors within the participants’ answers. This ratio represents the coverage of crowdsourcing, and is defined as follows:

RKRRParticipant answers that are known risksKnown risks

Thirteen (8+5) of the participants’ answers out of 16 known risk factors were derived from preceding studies and guidelines. This indicates that approximately 81% (13/16) of the hypotheses were reconfirmed by crowdsourcing. The number of new suitable hypotheses decreased steadily in later rounds.

#### Significant Known Risks Ratio

The SKRR is the ratio of significant risk factors within the ratio of known risk factors. This ratio represents the validity of crowdsourcing, and is defined as follows:

SKRRSignificant answers that are known risksParticipant answers that are known risks

Eight of the 13 known risk factors were statically significant, indicating that more than half (61%) of the risks had validity.

#### Significant Seed Risks Ratio

The SSRR is the ratio of significant risk factors within the known seed risk factors. This ratio also corresponds to the validity of the method, and is defined as follows:

SSRR = Significant seed risks / Seed risks = 4/4+4 = 0.50

Four of the 8 initial hypotheses (seed risk factors) were statically significant. Similar to the SKRR, this result also indicated that approximately half of the crowdsourced hypotheses had validity.

#### Significant Unknown Answer Ratio

The SUAR is the ratio of previously unknown significant associated factors within the participants’ significant answers. This ratio represents potential future topics for allergy research:

SUAR = Unknown significant answers / Significant answers = 53/53+8 = 0.86

This value (86%) indicates that there still remain many hypothesized associated factors that should be investigated in the future. In the context of the SSRR and SKRR results, half of these hypotheses may be valid risk factors.

### Types of Allergies

This study addressed 7 types of allergies: (1) asthma, (2) pollinosis, (3) allergic rhinitis, (4) atopic dermatitis, (5) food allergy, (6) drug hypersensitivity, and (7) sick building syndrome. Multimedia Appendix 2 shows the possible associated factors for overall allergies (Multimedia Appendix 2 A) and each of the 7 allergy types (Multimedia Appendix 2 B-H): 17 associated factors were identified for asthma (Multimedia Appendix 2 B); 31 associated factors were identified for pollinosis (Multimedia Appendix 2 C); 43 associated factors were identified for allergic rhinitis (Multimedia Appendix 2 D); 24 associated factors were identified for atopic dermatitis (Multimedia Appendix 2 E); 6 associated factors were identified for food allergies (Multimedia Appendix 2 F); 9 associated factors were identified for drug hypersensitivity (Multimedia Appendix 2 G), and 4 associated factors were identified for sick building syndrome (Multimedia Appendix 2 H).

Allergies are considered as, “exaggerated immune reactivity (hypersensitivity) to certain environmental substances (allergens) that normally have little effect on most people” and the, “hypersensitivity is established on initial exposure to the allergen (the sensitizing *dose*); subsequent exposure causes the hypersensitivity reaction” [22]. There are various types of allergies known to exist, and in this study we have focused on 7 of the most well-known allergy types in Japan. Allergies have become a serious national disorder in Japan, and the Japanese government has set up several boards for preventing allergies. The governmental guidelines of 2011 listed the major allergic symptoms, and we have used these symptoms in this study [23]. The current concepts, causes, and risk factors known for each specific allergy type are detailed below.

#### 1. Asthma

Asthma shows episodic reversible airway obstruction, increased bronchial reactivity, and airway inflammation. Causes of asthma can be divided into allergic and nonallergic etiologies. Allergies play an important role in asthma. For example, exposure to dust mites is associated with the development of asthma [24]. Mite and cockroach antigen exposures have been shown to increase asthma morbidity [24]. In this study, all types of possible asthmatic symptoms are included.

#### 2. Pollinosis

Pollinosis, commonly called *hay fever* (a term used in the past to describe farmers with symptoms occurring during *haying season* felt to be caused by the hay, but later determined to be caused by ragweed) is defined as the appearance of respiratory symptoms (rhinoconjunctivitis and/or asthma) resulting from the inhalation of pollen to which the individual is sensitized [25]. In this paper, the term is primarily applied to individuals reacting to Japanese red cedar, a seasonal form of allergic rhinitis. According to the Japanese government guideline of allergies, there are more than 60 kinds of possible major seasonal causes of pollinosis in Japan (eg, locust, Japanese cypress, birch, Japanese alder) [15]. In this study, we have included symptoms caused by all such possible allergens as *pollinosis*, and did not differentiate mono- or poly-sensitization during the season.

#### 3. Allergic Rhinitis

“Allergic rhinitis is a very common disorder that affects people of all ages, peaking in the teenage years. It underlies many complications, is a major risk factor for poor asthma control, and affects quality of life and productivity at work or school” [26]. Allergic rhinitis is frequently caused by exposure to perennial or seasonal allergens that exist in our indoor and outdoor environments [5]. Among the most common allergens, pollens (grass, trees, and weeds) are the predominant causes of seasonal allergic rhinitis [5]. House dust mites, pets, and molds are the major causes of perennial allergic rhinitis. However, in tropical and subtropical areas pollen may become a perennial allergen [5].

#### 4. Atopic Dermatitis

Atopic dermatitis is a skin condition characterized by a complex, heterogeneous pathogenesis, including skin barrier dysfunctions, allergy/immunology, and pruritus. When the skin barrier is disrupted, the skin is predisposed to being penetrated by external stimuli. Foreign antigens can be subdivided into two subsets by size: haptens (including metals) and protein antigens [27].

#### 5. Food Allergies

Food allergies are immunologically mediated adverse reactions to foods. Any food protein can trigger an allergic response, and allergic reactions to many foods have been documented. Many individuals have allergic reactions to food through mechanisms that are elusive [28,29].

#### 6. Drug Hypersensitivity

Drug hypersensitivity is an immune-mediated reaction to a drug. Symptoms range from mild to severe and include rash, anaphylaxis, and serum sickness. Symptoms and signs vary by patient and drug, and a single drug may cause different reactions in different patients. The most serious reaction is anaphylaxis; exanthema, urticaria, and fever are common. Fixed drug reactions—reactions that recur at the same body site each time a patient is exposed to the same drug—are uncommon [30].

#### 7. Sick Building Syndrome

Sick building syndrome, or building-related illness, is a heterogeneous group of disorders whose etiology is linked to the environment of modern airtight buildings. Such buildings are characterized by sealed windows and dependence on heating, ventilation, and air conditioning systems for the circulation of air. Most cases occur in nonindustrial office buildings, but cases can occur in apartment buildings, single-family homes, schools, museums, and libraries [31].

## Discussion

### Number of Diseases and Hypotheses

The number of participants with a particular disease (allergy) was found to be related to the number of hypotheses for that disease. Among the 7 types of allergies addressed in our questionnaire, many of the participants (n=130) reported suffering from allergic rhinitis, which produced the high number of significant possible associated factors (43 associated factors). Pollinosis, which had the highest number of sufferers (n=183), produced the second highest number of significant possible associated factors (31 associated factors). In contrast, sick building syndrome, reported by only 12 participants, produced 4 significant associated factors. Similarly, drug hypersensitivity, reported by only 13 participants, produced 9 significant associated factors. One of the possible reasons for this observation is that the high number of patients with an allergy may result in increased generation of hypotheses. This finding suggests that common diseases with many patients may be more suitable for crowdsourcing-based investigations.

### Genetic Factors Versus Environmental Factors

The results showed that most allergy types (with the exception of sick building syndrome) were associated with the genetic factor of *having family member(s) with an allergy*, which suggests a genetic basis for allergies. This, however, may also be interpreted to some degree as an environmental factor, as family members often share similar environments. In addition, participants who reported suffering from 5 types of allergies (except for atopic dermatitis and food allergies) were significantly associated with the condition of *often falling ill*, which may also suggest an environmental component to allergies. It is difficult to determine whether some environmental factors are the cause of an allergy, or its outcome.

### Disease Relationship: Pollinosis and Allergic Rhinitis

Among the 7 allergy types, pollinosis and allergic rhinitis shared many possible associated factors. These two diseases had 20 possible associated factors in common (out of 31 possible associated factors for pollinosis and 43 possible associated factors for allergic rhinitis), and also shared similar factors. Of the 183 participants (approximately 38%) with pollinosis and 130 participants (approximately 53%) with allergic rhinitis, 69 participants reported having both diseases. Interestingly, those with both diseases showed high ORs for exposure to emotionally stressful environments (*feeling temperamental or moody*; *feeling fatigued or stressed*; *having a traumatic and stressful experience*; *experiencing considerable environmental changes, such as moving and changing jobs*; and *having trouble with family relationships*) and food-related factors (*eating between meals* and *having a lot of ready-to-eat food/instant food*). These results corroborate the relationship between nasal-related allergies and emotional reactions that has been considered by recent allergy researches [32-34].

Conversely, it has been reported that preservatives can induce rhinitis (but this effect appears in very few cases associated with food), and this result somewhat indicates the relationship between eating habits and pollinosis/allergic rhinitis, which has been described in several previous studies [35,36].

### Asthma

Thirty-eight of the 502 participants reported suffering from asthma. The associated factor hypothesis of *often falling ill* (OR 6.81) may indicate that asthma itself implies a more delicate state of health. Some participants reported that they *have a harder time breathing during a typhoon* (OR 13.35), and this supports the theory that the typhoon season can worsen the asthma condition [37]. In addition, some individuals with asthma also experience *the onset of itching when touching certain metals* (OR 4.49). This result supports the relationship between asthma and metal allergy; especially as both may have been affected by the Asian Dust event [38]. Individuals with asthma appeared sensitive to their environment, such as *sensitive to smells* (OR 3.62), *sensitive to temperature change* (OR 2.88), as well as sensitivities to *the climate* and *typhoons*. Considering the asthma mortality rate for adults in Japan is still higher than in many Western countries [39], there is an urgent need to investigate the factors that contribute to the development of asthma. It was also noteworthy that those who *had measles during childhood* also tended to suffer from asthma. While a previous study reported that having childhood measles may reduce the diagnosis of asthma [40], another study claimed the opposite outcome [41]. This disparity requires further investigation.

### Pollinosis

Pollinosis is one of the most common diseases in Japan, and 183 of 502 participants in our study reported suffering from this disease. The hypothesized associated factor with the highest OR for pollinosis was that participants *sneeze often* (OR 3.64), but this is also one of the major symptoms of pollinosis. In the future, it will be necessary to determine causal directionality in such relationships, and identify whether a factor is a disease risk or a symptom/outcome. The results also indicated close relationships between this disease and stressful environments, including: *being in a very stressful environment* (OR 3.52); *experiencing considerable environmental changes, such as moving and changing jobs* (OR 3.26); *having a traumatic and stressful experience* (OR 2.56); *not feeling satisfied with life in general* (OR 3.41); *feeling temperamental or moody* (OR 2.08); and *having trouble with family relationships* (OR 1.85). These factors had higher ORs in pollinosis and allergenic rhinitis than in the other allergy types.

It is also notable that the participants with these two allergy types have experienced *eating food that had been prechewed by someone else* (OR 2.96) as a child, which used to be customary in some areas of Japan. This possible associated factor should be explored in greater depth, as this may indicate the possibility of a transmitted disease. In addition, *having family member(s) with an allergy* (OR 2.81) was significant in both of these types of allergies, indicating that genetic factors should also be investigated further. The hypothesis with the fifth highest OR was *being suntanned* (OR 3.46), and these participants may be more likely to spend time outdoors. Being suntanned and being exposed to pollen allergens may share a common primary factor, and further investigations are needed. Similarly, the results showed that individuals with pollinosis tended to be *habitually wearing facemasks* (OR 2.87), but this behavior may be used to avoid pollen exposure after the onset of symptoms. As stated earlier, the causal directionality of this relationship should be investigated further.

### Allergic Rhinitis

Allergic rhinitis is also a major common disease in Japan. In this study, 130 participants reported suffering from allergic rhinitis. As in pollinosis, the hypothesized associated factor that showed the highest OR for this allergy type was that participants *sneeze often* (OR 10.93), which is also a symptom of this allergy type. The results also indicated a possible relationship between *eating between meals* (OR 4.07) and allergic rhinitis. It is noteworthy that this allergy type showed similar possible associated factors with those of pollinosis. However, *being suntanned* was not significantly associated with allergic rhinitis, but *having birds as pets, and/or having close contact with birds* (OR 1.98) was unique to this allergy type. Although crowdworkers were assumed to have regarded pollinosis and allergic rhinitis as different diseases, we obtained similar possible associated factors. If the difference of the possible associated factors between these two diseases is meaningful, this should be validated externally in future work. Another novel finding was that *never been stung by a bee* (OR 2.73) and *habitually removing body hair* (OR 2.30) were shown to be related to allergic rhinitis, and both of these unique possible associated factors should be investigated further. Moreover, participants with allergic rhinitis showed significant relationships with the experience of *having been a target for bullying* as well as *living in apartments in higher floors when they were young*. This study, however, cannot distinguish between individuals who think they are allergic and those who have a nonallergic form of rhinitis (idiopathic rhinitis, nonallergic rhinitis with eosinophilia syndrome). A mechanism to remove these individuals from the grouping of allergic rhinitis needs to be established.

### Atopic Dermatitis

Fifty-nine participants reported suffering from atopic dermatitis in this study. The hypothesized associated factor that showed the highest OR was *suffering from atopic dermatitis as a child* (OR 30.25). This result shows that many individuals who suffered from this disease as a child continued to suffer as adults. Another hypothesized associated factor that showed a high OR was *often experiencing skin trouble (itching, rashes, etc.)* (OR 21.98), which is also a symptom of atopic dermatitis. A conspicuous and serious possible atopic dermatitis associated factor was *having sexual interactions since the participants were minors or in their teens* (OR 8.57). A similar hypothesis has been proposed by a previous study [42]. Discovery of this hypothesized associated factor may have been made possible by the high level of privacy afforded by crowdsourcing, which allowed the participants to honestly respond to highly private matters. Research that uses crowdsourcing may therefore have great potential for studies dealing with highly personal conditions.

Another possible associated factor was *not reading used books* (OR 6.32), which may be related to the fact that many participants with atopic dermatitis considered themselves to be *clean freaks* (OR 3.01); these individuals tend to avoid allergens as much as possible after they become aware of their allergy. Similarly, behaviors such as *preventing mite infestation (eg, on carpets, mattresses, beddings)* (OR 8.75), *being particular about using additive-free skin care products* (OR 5.77), and *wearing gloves when using detergents* (OR 5.48) may have been reactive countermeasures to their atopic dermatitis symptoms. Participants who reported *having close relationships with others who had allergies as a child* (OR 3.43) suggests that individuals with atopic dermatitis may have gathered with others who were also suffering allergic symptoms, indicating some psychological care for children with atopic dermatitis. This associated factor may have a possible influence on character development. In addition, those with atopic dermatitis showed some unique associated factors such as *not feeling sick when traveling abroad* (OR 9.75) and *not using contact lenses or glasses* (OR 3.97). Further investigation is needed for these factors.

### Food Allergy

Twenty-six of 502 participants reported having a food allergy, and the hypothesized associated factor of *having family member(s) with an allergy* (OR 3.71) showed a relatively high OR in these individuals, and in those with drug hypersensitivity (OR 16.21). This result may suggest a strong genetic influence in these allergy types. However, between these two allergy types, only food allergies showed a significant relationship with *suffering from atopic dermatitis as a child* (OR 4.69), which supports the findings from previous studies that indicated a relationship between these two allergy types [28,43]. The low number of participants who reported having drug hypersensitivity may also have influenced these findings (we did not observe any relationship with *suffering from atopic dermatitis as a child*), and further studies that compare these two particular allergy types are needed to shed light on this topic.

Those with a food allergy were associated with *often suffering from hives* (OR 3.44), which may also be one of their symptoms. Another possible associated factor was *frequent cleaning of the house* (OR 5.22), which may be a behavioral response to their allergy, as individuals with food allergies frequently attempt to remove allergens from their house after they become aware of their disease. Conversely, this result may support the so-called *hygiene hypothesis*, which states that childhood exposure to allergens can reduce susceptibility to allergies. However, two other possible associated factors identified in our study may be contrary to the hygiene hypothesis: *played at vacant lots during childhood* (OR 5.71) and *played in mountains and bushes during childhood* (OR 3.45). Further investigations should therefore be conducted on the relationship between food allergies and the hygiene hypothesis.

### Drug Hypersensitivity

Thirteen of 502 participants reported suffering from drug hypersensitivity, which was half the number of those with a food allergy. Similar to food allergies, this type of allergy had a significant relationship with the genetic factor of *having family member(s) with an allergy* (OR 16.21). However, unlike food allergies, drug hypersensitivity showed more environmental factors as possible associated factors, including: *often wearing cosmetics* (OR 5.07); *experiencing considerable environmental changes, such as moving and changing jobs* (OR 5.22); and *owning pets or having general contact with animals* (OR 4.09). The relationship between drug hypersensitivity and cosmetics should be investigated, especially on the specific types of cosmetics that may have influenced this allergy. In addition, those who experienced *often falling ill* (OR 0.96) and who *often caught colds during childhood* (OR 3.65) also showed significant relationships with this allergy type. Such individuals may have increased exposure to medications, which may be an associated factor for drug hypersensitivity [44].

### Sick Building Syndrome

Twelve of 502 participants reported suffering from sick building syndrome, making this the least represented allergy in our study sample. This allergy type also produced the fewest number of hypotheses. The hypothesized associated factor with the highest OR was *having felt sick after changing wall paper* (OR 48.50). This, however, can be one of the symptoms of this allergy type and is not considered to be a novel associated factor. The factor of *often falling ill* (OR 7.37) had the third highest OR for this allergy type. This finding may indicate possible allergies due to exposure to medications and a symptom resulting from this allergy. Similarly, other hypothesized associated factors that were significantly associated with sick building syndrome were *using cotton towels* (OR 12.92) and *habitually wearing facemasks* (OR 5.57), which are both very likely to be countermeasures against this allergy. As a result, this research could not identify any useful hypotheses for sick building syndrome, which suggests that research using crowdsourcing may have problems with small sample sizes.

### Optimal Stopping of the Algorithm

There were difficulties in determining the optimal stopping of the algorithm (questionnaire rounds) because more data would ostensibly give rise to more accurate results with negligible increases in cost. A strong indication for the optimal end time was the number of new hypothesized associated factors. In this experiment, Round 1 produced 54 hypotheses, Round 2 produced 42 hypotheses, Round 3 produced 33 hypotheses, and Round 4 produced 28 hypotheses. This steady decrease indicated that there may be few novel hypotheses generated in further iterations of the algorithm. Other indications for optimal stopping of the algorithm included the steady decrease in the number of significant hypotheses and the number of hypotheses that were identical to those previously proposed. In the near future, these aforementioned statistics may support the development of an optimal stopping theory for studies such as this.

### Limitations

This study had several limitations. A major limitation is the possible sampling bias due to the use of Web-based participants. Age, gender, and other background characteristics may differ from the general population, and thereby reduce the representativeness of our findings. While Internet usage for people aged <50 years is over 90% in Japan, half of those aged >70 years do not have significant access to the Internet [8]. This study used one crowdsourcing company (Yahoo! Japan [16]) for sampling, and the users of other crowdsourcing companies were not included. This limitation may have resulted in sampling error. Current methodologies make it difficult to avoid such biases, and new techniques are needed.

Another limitation is the difficulty in determining the causal directionality of relationships. For example, one of the hypothesized associated factors for pollinosis is *sneeze often* but this can be a symptom of pollinosis itself. In fact, the new hypothesis generated in this study contained many of these types of associated facts. Ultimately, if we have already obtained enough knowledge on a disease, we could classify the results in three ways: (1) a symptom/outcome of the allergy (eg, *sneeze often*); (2) a behavioral response to the allergy (eg, *clean your house frequently*); and (3) a potential causal relationship, which is a target of this study. However, such classifications tend to be subjective and difficult to distinguish. For example, several studies have indicated that environments that are *too clean* during childhood may cause a risk of allergies [45,46]. Such a hypothesis causes difficulty for classification, partly because the terminology and granularity of the descriptions are different between the crowdsourced results and existing known risks. For example, a crowdsourced result, “ *Do you often undergo illnesses?* ” does not match the existing findings, because some words, such as *often* and *illnesses*, are not well-defined. Crowdsourced results often contain various vague words. Further studies are required to fill this gap, which makes the obtained hypotheses suitable for external validity.

Furthermore, most of the crowdsourcing participants lack in-depth knowledge about allergies, presumably lack detailed medical knowledge, and the concept of allergies may be misunderstood by some individuals. To deal with this problem, we could use an alternative questionnaire to ask, “ *Have you ever been diagnosed by a physician with X?* ” which is based on a physician’s expertise. This study has room to improve the credibility of participants’ answers.

Finally, it was labor-intensive and costly to remove inadequate candidate hypotheses generated by the participants. Many participants submitted the hypotheses individually, and we found that many hypotheses overlapped with one another. To avoid these overlaps, we had to manually check the newly proposed questions, which required a great deal of effort and time. For every 100 questions, this checking process incurred the cost equivalent to hiring one person per day (on average). In the near future, advances in natural language processing techniques are expected to contribute to easing the demands of this labor-intensive and potentially costly process. This study cannot provide medical diagnoses for individual participants (ie, it is not possible to definitively tell an individual that they have allergic rhinitis or a food allergy). Detailed diagnoses and precise medical studies are awaited to determine the novel medically-qualified allergy risk factors.

### Conclusions

The aim of this study was to utilize and apply a Web crowdsourcing service to collect and test hypotheses for possible allergy-associated factors. We crowdsourced for unknown allergy-associated factors for seven different allergies, and calculated their ORs. This study was unique in that it also asked the participants to generate original hypotheses to allow the general public to contribute to identifying possible causes of allergies that even an experienced physician may have difficulties conceiving. This task identified more than 157 new hypotheses, including 53 significantly associated factors that were previously unknown. These novel factors warrant further in-depth investigation, and clinical trials should also be conducted in the future to validate these hypotheses.

## Appendix

Multimedia Appendix 1 The questionnaire used in the crowdsourcing survey.

Multimedia Appendix 2 Questions (hypothesized associated factors) with significant odds ratios (95% CI: over 1.0).

## Abbreviations

OR: odds ratio
RKRR: Rediscovered Known Risk Ratio
SKRR: Significant Known Risks Ratio
SSRR: Significant Seed Risks Ratio
SUAR: Significant Unknown Answer Ratio
