# Supplementary material for: Crowdsourced Identification of Possible Allergy-Associated Factors: Automated Hypothesis Generation and Validation Using Crowdsourcing Services
Source: JMIR Res Protoc. 2017 May 16;6(5):e83. doi: 10.2196/resprot.5851 (PMC5449648; doi:10.2196/resprot.5851)
Supplement: Multimedia Appendix 1 [file resprot_v6i5e83_app1.pdf]

Multimedia Appendix 1. The questionnaire used in the crowdsourcing survey.

Profile Questions

P1) Age: ◯ 10-19 ◯ 20s ◯ 30s ◯ 40s ◯ 50s ◯ 60s and/or above (__)

P2) Sex: ◯ Male ◯ Female

P3) Marital Status: ◯ Single ◯ Married

P4) Number of siblings: ◯ Only child ◯ 1 ◯ 2 ◯ 3 ◯ More (__)

P5) Do you have any allergies? :

◻ Asthma ◻ Pollinosis ◻ Allergic rhinitis ◻ Atopic dermatitis ◻ Food allergy

◻ Drug hypersensitivity ◻ Sick building syndrome ◻ None

Risk Questions (Seed)

Q1) Do(es) any of your family member(s) have an allergy?

◻ Father ◻ Mother ◻ Elder brother ◻ Younger brother ◻ Elder sister ◻ Younger sister ◻ Son

◻ Daughter ◻ Other (__)

Q2) Do you wear any piercings?

◯ Yes ◯ No ◯ I used to.

Q3) Do you have any decayed teeth?

◯ Yes, and had them treated (about __ years ago). ◯ Yes, but I haven't gotten them treated. ▯ ◯ Never.

Q4) Do you have any false teeth?

◯ Yes. ◯ No.

Q5) Where were you born?

◯ Industrial area ◯ Urban area ◯ Suburb/countryside

Q6) When you were a child, did you often catch cold?

◯ Yes. ◯ No.

Q7) Do (Did) you have a good relationship with your family?

◯ Yes. ◯ I had/have a lot of stress.

Q8) Are you temperamental or moody?

◯ Yes. ◯ No.

Risk Questions (Proposed)

Q9) Do you live in a newly built house?

◯ Yes. ◯ No.

Q10) ……

Novel Risk-Proposal Question

R1) Please submit some of your own questions that you feel may help to detect the cause of an allergy.

(e.g.) Were you *ing when you were a child? [Please be as specific as possible.]

Were/Do/Did you (□□□□□□□□□□□□□□□□□□□□□□□□□) ?

[TEXT BOX] [TEXT BOX] [TEXT BOX] [TEXT BOX] [TEXT BOX]

○ Indicates a radio box (Single choice)

□ Indicates a check box (Multiple choice)

(Top) **Profile questions** (Middle) **Risk questions** (Bottom) **Novel risk-proposal question**
