# Supplementary material for: Crowdsourced Identification of Possible Allergy-Associated Factors: Automated Hypothesis Generation and Validation Using Crowdsourcing Services
Source: JMIR Res Protoc. 2017 May 16;6(5):e83. doi: 10.2196/resprot.5851 (PMC5449648; doi:10.2196/resprot.5851)
Supplement: Multimedia Appendix 2 [file resprot_v6i5e83_app2.pdf]

Multimedia Appendix ###. Questions (hypothesized associated factors) with significant odds ratios (95% CI: over 1.0).

(A) Total

Do(es) any of your family member(s) have an allergy?
When you were a child, did you often catch cold?
Are you temperamental or moody?
Do you often take medications and/or supplements? Or do you habitually take any medications?
Do you feel itchy when you touch some metals?
Do you often use cosmetics?
Do you have any (furry) pets and/or animals around you?
Do you clean your house frequently?
Do you consider yourself a “clean freak”?
Do you habitually wash your hands and gargle?
When you were a child, did you often go and play in mountains and bushes?
Do you often experience skin troubles? (E.g. itchiness, skin rash, etc.)
Do you feel itchy when you are bitten by bugs such as mosquitos?
When you were a child, did you have atopic dermatitis?

Do you often have hives?
Have you ever been hospitalized when you were a child?
Do you often fall ill?
Have you ever had food poisoning?
Are you constipated often?
Do you often sneeze or have an itchy nose?
When you were a child, did you play at vacant lots?
Are you suntanned?
Are there fields of rice and other crops around where you live/work?
Do you sometimes keep eating the same food over and over?
Do you eat between meals?
Do you regularly wear face masks?
Are you frequently exposed to dusty conditions?
Do you have certain smell or fragrances that you can sense better than other people?
Do you keep birds as pets, or do you have close contact with birds?
Do you have a lot of stuffed toys?

Are you taking any anti-mildew measures?
Do you frequently use disinfection products?
When you were a child, did you often go and play in rivers or ponds?
Do you often feel tired?
Did you have measles when you were a child?
Did you often have stomach aches when you were a child?
Have you had any trouble after getting injections?
Does your skin feel itchy when it is dry?
Do you sleep in a bed? (cf. sleeping in a futon)
Did you live in an apartment in higher floors when you were a child?
Do you frequently eat ready-to-eat food (instant food)?
Do you eat out often?
Do you usually have breakfast?
Do you feel fatigued or stressed these days?
Do you have any traumatic and stressful experiences?
Have you experienced considerable environmental changes, such as moving and changing jobs?

Have you had food that had been pre-chewed by someone else? (E.g. by your mother when you were a baby)
Are you more sensitive to temperature changes when compared to other people?
Does your physical condition change as your body temperature changes? (E.g. after sports, etc.)
Is (Are) there any particular place(s) where you feel sick each time that you go?
Are you particular about using additive-free skin care products?
Do you often ventilate and/or dehumidify using an air-conditioner or any other equipment?
Do you habitually remove your body hair?
Do (Did) you often have acne on your skin?
Have you ever felt sick after changing wallpaper?
Is (Are) there any kind(s) of food you have been avoiding since your childhood?
Do you wear gloves when you use detergent?
Do you feel sick when the seasons change?
Do you carry your handkerchief with you when you go out?
Are you working to prevent mite infestations? (E.g. on carpets, mattresses, or bedding)
Does your skin swell when it gets scratched?
Do you often have eye discharge?

Have you ever been a target of bullying?
Do you have trouble breathing during typhoons?
When you were a child, did you have close relationships with others with allergies?
Did you have sexual interactions as a minor or in your teens?
Do you develop chapped hands when using detergent?
Do you often wear headphones?
Do (Did) you have a bad relationship with your family?
Do (Did) you feel sick when you go (went) abroad?
Do you use cotton towels?
Do you wear contact lenses or glasses?
Are you dissatisfied with your life in general at present?
Do you habitually read used books?
Have you ever been stung by a bee?

(B) Asthma

Question (Hypothesis)	Allergy Positive		Allergy Negative		Odds Ratio	95%CI (Lower bound)
	YES	NO	YES	NO		
Do you often undergo illnesses?	22	9	98	273	6.81	3.03
Do you have trouble breathing when typhoon comes?	3	5	4	89	13.35	2.33
Do you often have hives?	16	15	71	300	4.51	2.13
Do you feel itchy when you touch some metal?	9	22	31	340	4.49	1.90
Do you feel itchy when you are bit by bugs like mosquitos?	6	1	27	66	14.67	1.68
Do(es) any of your family member(s) have allergy?	27	11	193	271	3.45	1.67
When you were a child, did you have atopic dermatitis?	10	21	47	324	3.28	1.46
Do you have much skin troubles (e.g. itchiness, skin rash, etc.)?	25	6	202	169	3.49	1.40
Do you often sneeze or have itchy nose?	25	6	202	169	3.49	1.40

Have you had any trouble after getting injections?	6	18	22	256	3.88	1.40
Do you have certain smell or fragrances that you can sense better than other people?	18	6	126	152	3.62	1.39
Are you more sensitive to the temperature change compared to other people?	14	10	91	187	2.88	1.23
Does your physical condition change as your body temperature changes? (e.g. after sports, etc.)	13	11	87	191	2.59	1.12
When you were a child, did you often catch cold?	17	14	129	242	2.28	1.09
Did you have measles when you were a child?	19	5	156	122	2.97	1.08
Do you habitually wear face masks?	9	22	55	316	2.35	1.03
Have you ever been hospitalized when you were a child?	7	6	50	137	3.20	1.02

(C) Pollinosis

**Question (Hypothesis)**	**Allergy Positive**		**Allergy Negative**		**Odds Ratio**	**95%CI (Lower bound)**
	**YES**	**NO**	**YES**	**NO**		

Do you often sneeze or have itchy nose?	114	38	113	137	3.64	2.33
Do you feel fatigued or stressed these days?	94	22	102	84	3.52	2.04
Do(es) any of your family member(s) have allergy?	110	74	110	208	2.81	1.93
Have you experienced much environmental changes, such as moving and changing jobs?	39	77	25	161	3.26	1.84
Are you sun-tanned?	51	18	59	72	3.46	1.83
Do you habitually wear face masks?	38	114	26	224	2.87	1.66
Have you had food that was masticated by someone else? (e.g. by your mother when you were a baby)	45	26	48	82	2.96	1.62
Do you have any traumatic and stressful experience?	71	45	71	115	2.56	1.59
Do you sometimes keep eating the same food over and over?	90	17	128	66	2.73	1.50
Are you dissatisfied with your overall aspects of life now?	33	12	25	31	3.41	1.46
Are you temperamental or moody?	108	76	129	189	2.08	1.44
Have you ever had food poisoning?	108	44	135	115	2.09	1.36

Do you eat between meals?	133	19	188	62	2.31	1.32
Do you have certain smell or fragrances that you can sense better than other people?	68	48	76	110	2.05	1.28
Do you often have eye mucus?	26	19	18	38	2.89	1.28
Do (Did) you have a bad relationship with your family?	79	105	92	226	1.85	1.26
Is (Are) there any kind(s) of food you have been avoiding since your childhood?	49	34	47	72	2.21	1.25
Do you have much skin troubles (e.g. itchiness, skin rash, etc.)?	100	52	127	123	1.86	1.23
Is (Are) there any particular place(s) where you feel sick each time you go?	35	81	32	154	2.08	1.20
Are you taking any mildew-proof measures?	53	63	57	129	1.90	1.18
Do you have any (hairy) pets and/or animals around you?	79	73	95	155	1.77	1.17
Did you often have stomach ache when you were a child?	56	60	62	124	1.87	1.16
Do you often wear headphones?	24	21	17	39	2.62	1.16
Do you usually have breakfast?	102	14	143	43	2.19	1.14
Do you have your skin swollen when it gets scratched?	69	47	83	103	1.82	1.14

Do you frequently have ready-to-eat food (instant food)?	72	44	88	98	1.82	1.14
Do you often undergo illnesses?	56	96	64	186	1.70	1.10
Do you habitually wash your hands and gargle?	121	31	174	76	1.70	1.06
Have you had any trouble after getting injections?	16	100	12	174	2.32	1.06
Do you clean your house frequently?	102	50	140	110	1.60	1.05
Are there fields of rice and other crops around you?	96	56	131	119	1.56	1.03

(D) Allergic rhinitis

**Question (Hypothesis)**	**Allergy Positive**		**Allergy Negative**		**Odds Ratio**	**95%CI (Lower bound)**
	**YES**	**NO**	**YES**	**NO**		
Do you often sneeze or have itchy nose?	96	11	131	164	10.93	5.62
Do you eat between meals?	99	8	222	73	4.07	1.89
Do you have any traumatic and stressful experience?	55	27	87	133	3.11	1.83

Do you have certain smell or fragrances that you can sense better than other people?	55	27	89	131	3.00	1.76
Do(es) any of your family member(s) have allergy?	80	50	140	232	2.65	1.76
Do (Did) you often have pimples on your skin?	24	7	30	40	4.57	1.74
Do you have your skin swollen when it gets scratched?	56	26	96	124	2.78	1.63
Have you experienced much environmental changes, such as moving and changing jobs?	29	53	35	185	2.89	1.62
Do (Did) you have a bad relationship with your family?	64	66	107	265	2.40	1.59
Do you have much skin troubles (e.g. itchiness, skin rash, etc.)?	77	30	150	145	2.48	1.54
Do you feel itchy when you are bit by bugs like mosquitos?	14	11	19	56	3.75	1.46
Are you temperamental or moody?	80	50	157	215	2.19	1.46
Are you sleeping in bed? (Not in Futon)	62	20	121	99	2.54	1.43
Is there much dust around you?	42	20	62	77	2.61	1.39
Do you often undergo illnesses?	45	62	75	220	2.13	1.34

Does your physical condition change as your body temperature changes? (e.g. after sports, etc.)	38	44	62	158	2.20	1.30
When you were a child, did you often catch cold?	52	55	94	201	2.02	1.29
When you were a child, did you have atopic dermatitis?	24	83	33	262	2.30	1.28
Do you feel sick when season changes?	42	9	94	57	2.83	1.28
Have you been a target of bullying(s)?	13	7	28	53	3.52	1.26
Do you often ventilate and/or dehumidify by using air-conditioner or any other equipment?	32	19	62	89	2.42	1.26
Is (Are) there any particular place(s) where you feel sick each time you go?	27	55	40	180	2.21	1.24
Do you have any (hairy) pets and/or animals around you?	59	48	115	180	1.92	1.23
Did you live in the upper floors when you were small?	11	71	11	209	2.94	1.22
Are you taking any mildew-proof measures?	40	42	70	150	2.04	1.22
Do you feel fatigued or stressed these days?	63	19	133	87	2.17	1.21
Do you have birds, or are you in the environment to interact with them?	39	43	69	151	1.98	1.18

Are you more sensitive to the temperature change compared to other people?	38	44	67	153	1.97	1.17
Are you constipated often?	28	28	45	99	2.20	1.17
Have you had any trouble after getting injections?	13	69	15	205	2.57	1.17
Do you habitually wear face masks?	25	82	39	256	2.00	1.14
Do you habitually epilate your body hair?	17	34	27	124	2.30	1.12
Have you ever been stung by bees?	10	21	39	30	2.73	1.12
Do you often take medications and/or supplements? Or do you habitually take any medication?	11	14	16	59	2.90	1.11
Do you carry your handkerchief with you when you go out?	39	12	89	62	2.26	1.10
Have you had food that was masticated by someone else? (e.g. by your mother when you were a baby)	36	26	57	82	1.99	1.09
Do you have a lot of stuffed dolls?	37	45	69	151	1.80	1.07
Do you frequently have ready-to-eat food (instant food)?	52	30	108	112	1.80	1.07
Do you often feel tired?	45	17	79	60	2.01	1.05

Do you eat out often?	25	37	36	103	1.93	1.03
Do you frequently use disinfection goods?	34	48	64	156	1.73	1.02
Are you particular about using additive-free skin care cosmetics?	16	35	27	124	2.10	1.02
Do you clean your house frequently?	73	34	169	126	1.60	1.00

(E) Atopic dermatitis

Question (Hypothesis)	Allergy Positive		Allergy Negative		Odds Ratio	95%CI (Lower bound)
	YES	NO	YES	NO		
When you were a child, did you have atopic dermatitis?	33	15	24	330	30.25	14.47
Do you have much skin troubles (e.g. itchiness, skin rash, etc.)?	46	2	181	173	21.98	5.26
Does using of detergent chap your hands?	11	2	23	65	15.54	3.20
Do you feel itchy when your skin is dry?	33	2	151	116	12.68	2.98

Do (Did) you feel sick when you go (went) abroad?	1	12	39	48	9.75	1.21
Do you feel itchy when you are bit by bugs like mosquitos?	10	3	23	64	9.28	2.34
Are you coping with mites? (e.g. on carpets, mattresses, bedding)	9	4	18	70	8.75	2.42
Did you have sexual interaction since under-age (or since in your teens) ?	6	7	8	80	8.57	2.31
Do you habitually read used-books?	2	24	60	114	6.32	1.44
Are you particular about using additive-free skin care cosmetics?	12	10	31	149	5.77	2.29
Do you wear gloves when you use detergent?	8	14	17	163	5.48	2.01
Do you often have hives?	24	24	63	291	4.62	2.47
Do you wear contact lenses or glasses?	6	7	68	20	3.97	1.20
Do you feel itchy when you touch some metal?	12	36	28	326	3.88	1.82
When you were a child, did you often catch cold?	7	4	28	61	3.81	1.03
When you were a child, did you often go and played in rivers or ponds?	16	6	75	104	3.70	1.38

Does your physical condition change as your body temperature changes? (e.g. after sports, etc.)	21	14	79	188	3.57	1.73
When you were a child, did you have close relationship with others with allergies?	8	5	28	60	3.43	1.03
Do you have your skin swollen when it gets scratched?	26	9	126	141	3.23	1.46
Are you a clean-freak?	15	20	53	213	3.01	1.45
Do(es) any of your family member(s) have allergy?	38	21	182	261	2.59	1.47
Do you often sneeze or have itchy nose?	35	13	192	162	2.27	1.16
Are you more sensitive to the temperature change compared to other people?	18	17	87	180	2.19	1.08
When you were a child, did you often go and played in mountains and bushes?	21	18	93	169	2.12	1.08

(F) Food allergy

**Question (Hypothesis)**	**Allergy Positive**		**Allergy Negative**		**Odds Ratio**	**95%CI (Lower bound)**
	**YES**	**NO**	**YES**	**NO**		

When you were a child, did you have atopic dermatitis?	7	10	50	335	4.69	1.71
Do(es) any of your family member(s) have allergy?	19	7	201	275	3.71	1.53
Do you often have hives?	8	9	79	306	3.44	1.29
Do you clean your house frequently?	15	2	227	158	5.22	1.18
When you were a child, did you play at vacant lot?	3	3	14	80	5.71	1.05
When you were a child, did you often go and played in mountains and bushes?	8	4	106	183	3.45	1.02

(G) Drug hypersensitivity

**Question (Hypothesis)**	**Allergy Positive**		**Allergy Negative**		**Odds Ratio**	**95%CI (Lower bound)**
	**YES**	**NO**	**YES**	**NO**		
Do(es) any of your family member(s) have allergy?	12	1	208	281	16.21	2.09
Do you often undergo illnesses?	8	4	112	278	4.96	1.47

Do you often wear cosmetics?	6	3	54	137	5.07	1.22
Do you often have hives?	6	6	81	309	3.81	1.20
Have you experienced much environmental changes, such as moving and changing jobs?	4	3	60	235	5.22	1.14
Do you have any (hairy) pets and/or animals around you?	9	3	165	225	4.09	1.09
When you were a child, did you often catch cold?	8	4	138	252	3.65	1.08
Is (Are) there any particular place(s) where you feel sick each time you go?	4	3	63	232	4.91	1.07
Does your physical condition change as your body temperature changes? (e.g. after sports, etc.)	5	2	95	200	5.26	1.00

(H) Sick building syndrome

**Question (Hypothesis)**	**Allergy Positive**		**Allergy Negative**		**Odds Ratio**	**95%CI (Lower bound)**
	**YES**	**NO**	**YES**	**NO**		
Have you ever felt sick after changing the wallpaper?	1	1	2	97	48.50	2.17

Do you often undergo illnesses?	6	2	114	280	7.37	1.47
Do you habitually wear face masks?	4	4	60	334	5.57	1.36
Do you use cotton towels?	1	2	84	13	12.92	1.09
